# From Geometry of Hamiltonian Dynamics to Topology of Phase Transitions: A Review

**DOI:** 10.3390/e26100840

**Published:** 2024-10-05

**Authors:** Giulio Pettini, Matteo Gori, Marco Pettini

**Affiliations:** 1 Dipartimento di Fisica, Università di Firenze, and I.N.F.N., Sezione di Firenze, via G. Sansone 1, I-50019 Sesto Fiorentino, Italy; pettini@fi.unifi.it; 2 Department of Physics and Sciences of Materials, University of Luxembourg, L-1511 Luxembourg, Luxembourg; gori6matteo@gmail.com; 3 Aix-Marseille Univ, CNRS, Université de Toulon, 13288 Marseille, France; 4 Centre de Physique Théorique, 13288 Marseille, France; 5 Quantum Biology Lab, Howard University, Washington, DC 20059, USA

**Keywords:** statistical mechanics, phase transitions, Hamiltonian dynamics, Riemannian geometry, differential topology, 05.20.Gg, 02.40.Vh, 05.20.- y, 05.70.- a

## Abstract

In this review work, we outline a conceptual path that, starting from the numerical investigation of the transition between weak chaos and strong chaos in Hamiltonian systems with many degrees of freedom, comes to highlight how, at the basis of equilibrium phase transitions, there must be major changes in the topology of submanifolds of the phase space of Hamiltonian systems that describe systems that exhibit phase transitions. In fact, the numerical investigation of Hamiltonian flows of a large number of degrees of freedom that undergo a thermodynamic phase transition has revealed peculiar dynamical signatures detected through the energy dependence of the largest Lyapunov exponent, that is, of the degree of chaoticity of the dynamics at the phase transition point. The geometrization of Hamiltonian flows in terms of geodesic flows on suitably defined Riemannian manifolds, used to explain the origin of deterministic chaos, combined with the investigation of the dynamical counterpart of phase transitions unveils peculiar geometrical changes of the mechanical manifolds in correspondence to the peculiar dynamical changes at the phase transition point. Then, it turns out that these peculiar geometrical changes are the effect of deeper topological changes of the configuration space hypersurfaces $\sum_{v}=V_{N}^{-1}(v)$ as well as of the manifolds ${\left\{M_{v}=V_{N}^{-1}((-\infty ,v])\right\}}_{v}\in R$ bounded by the ∑_v_. In other words, denoting by vc the critical value of the average potential energy density at which the phase transition takes place, the members of the family ${\left\{\sum_{v}\right\}}_{v<v_{c}}$ are not diffeomorphic to those of the family ${\left\{\sum_{v}\right\}}_{v>v_{c}}$; additionally, the members of the family ${\left\{M_{v}\right\}}_{v>v_{c}}$ are not diffeomorphic to those of ${\left\{M_{v}\right\}}_{v>v_{c}}$. The topological theory of the deep origin of phase transitions allows a unifying framework to tackle phase transitions that may or may not be due to a symmetry-breaking phenomenon (that is, with or without an order parameter) and to finite/small *N* systems.

## 1. Introduction

Statistical physics has been devised by its founding fathers, Boltzmann and Gibbs, to predict the macroscopic physical properties of systems composed of a large number of atoms or molecules by getting rid of the knowledge of microscopic dynamics.

On the basis of the knowledge of the interatomic or intermolecular forces acting among a many-body system with a large number of degrees of freedom, the Gibbs ensemble formulation of statistical mechanics allows us to derive all the macroscopic equilibrium properties of such a system. Moreover, there is a variety of phenomena (also making up part of our common daily experience) such as the condensation of a gas or the solidification of a liquid—in general, a change in the state of aggregation of matter—whose explanation is naturally expected within the framework as statistical mechanics. This last topic is part of a broad and interesting field that includes a wealth of collective phenomena: *phase transitions*. In nature, at very different scales of space and energy, phase transition phenomena are ubiquitous. Usually, phase transitions are related to a spontaneous symmetry breaking [1,2]. However, this is not an all-encompassing framework because many physical systems do not fit this scheme and undergo a phase transition in the absence of symmetry breaking. Furthermore, while the Yang–Lee [3,4] and Dobrushin–Lanford–Ruelle [5] theories require the limit N→∞ (thermodynamic limit) to mathematically describe a phase transition, transition phenomena in small-N systems are particularly relevant in many contemporary problems and are at odds with this theoretical framework.

A new insight into the foundations of statistical mechanics became available at the dawning of the computer era with the pioneering work of E. Fermi, J. von Neumann and S. Ulam [6] through the “long time” numerical solution of the equations of motion of a set of interacting particles. Since then, a vast literature on the dynamical phenomenology of many -particle Hamiltonian systems has been produced, and, among other phenomena, phase transitions have been also investigated from the microscopic dynamical viewpoint [7,8,9,10,11].

The novelty of the theoretical proposal discussed here comes from the combination of the study of phase transitions from the point of view of Hamiltonian dynamics with the identification of the natural motions of a Hamiltonian system with the geodesics of properly defined Riemannian manifolds [12,13,14,15,16], hence the possibility to deepen our understanding of the relation between the macroscopic physics of many-particle systems with their microscopic dynamical counterpart. In fact, after the Riemannian geometrization of dynamics, we can naturally wonder whether there is a specific geometrical counterpart occurring to these mechanical manifolds when a Hamiltonian system displays a phase transition: in other words, if and how the appearance of a phase transition can be “geometrically read”. This is where topology comes into play and, in a sense to be specified, it is found that for a large class of systems, a phase transition can occur *only if* the mentioned geometric changes are the product of suitable topological changes of certain submanifolds of the configuration space. The remarkable consequence from a theoretical point of view is that the singular behaviors of thermodynamic observables at the critical point of a phase transition follow from a phenomenon which is independent of the statistical measures. The topological origin of phase transitions is a necessary but not sufficient condition as is proven by two theorems and by the study of exactly solvable models.

The paper is organized as follows. In Section 2, we review how classical Hamiltonian dynamics is “translated” into geometrical terms. In Section 3, the dynamical and geometric counterparts of phase transitions are considered. In Section 4, how this leads to the topological theory of phase transitions is then discussed. Finally, in a discussion section, an overview is given about present and future prospective applications in both classical and quantum systems.

## 2. Riemannian Geometric Formulation of Hamiltonian Dynamics

By a *natural* Hamiltonian system, it is meant a system whose kinetic energy is a quadratic form in the velocities. Any Newtonian system, that is, a system of particles interacting by forces derived from a potential, belongs to this class. The solutions of Newton equations are trajectories of the configuration space that can be identified with the geodesics of an appropriate Riemannian manifold. This variational formulation of the dynamics is at the basis of this classic result. In fact, after the least action principle, the natural movements of a Hamiltonian/Newtonian system are the extrema of the Hamiltonian action S

(1) S=∫Ldt,

where L is the Lagrangian function of the system, and on the other side, the geodesics of a Riemannian manifold are the extrema of the length functional

(2) ℓ=∫ds,

where s is the arc length parameter. By establishing the link between length and action, thanks to an appropriate choice of metric, the identification of geodesics of an appropriate Riemannian manifold with the physical trajectories naturally follows.

The added value of the geometric formulation of Hamiltonian/Newtonian dynamics consists in the understanding of the origin of deterministic chaos in these systems together with the possibility of analytically computing the largest Lyapunov exponent, the paradigmatic indicator of the presence of chaos and measure of its strength.

Dating back to the 1940s, N.S. Krylov [12] tried to exploit the Riemannian geometrization of Hamiltonian dynamics to account for the relaxation to the equilibrium of many-particle systems through the dynamical instability later called *deterministic chaos*. The subsequent attempts to explain the origin of chaos in Newtonian/Hamiltonian dynamical systems by resorting to the mentioned Riemannian framework invariably failed. These failures were the consequence of the unquestioned hypothesis according to which chaos can arise only from the hyperbolicity of the mechanical manifolds. To the contrary, as it will be discussed below, numerical experiments have revealed another mechanism (parametric resonance due to curvature variability along the geodesics) entailing the chaotic instability of geodesic flows associated with physically significant Hamiltonians. The instability of the geodesics of mechanical manifolds, and therefore the study of chaos, makes use of the Jacobi–Levi–Civita equation for the geodesic spread to work out an analytic formula for the largest Lyapunov exponent. Applied to some models, this analytic formula allows to find a strikingly good agreement between theoretical predictions and numerical values of the largest Lyapunov exponent computed by means of the standard tangent dynamics equation.

### 2.1. The Jacobi Metric in Configuration Space

There are several possible choices for the ambient space and its metric to formulate the Riemannian geometrization of classical dynamics. Among other possible choices, the most known one since the nineteenth century is the so-called Jacobi metric on the configuration space of a given system. In fact, Krylov’s work was performed in this geometric framework. Let us consider systems described by a Lagrangian of the form

(3) $$L\left(\dot{q},q\right)=\frac{1}{2}a_{ij}\left(q\right){\dot{q}}^{i}{\dot{q}}^{j}-V\left(q\right),$$

where

(4) $${\dot{q}}^{i}\left(t\right)=\frac{dq^{i}}{dt}\left(t\right)$$

and $a_{ij}$ are the components of the kinetic energy matrix, and V(q) is the potential energy. It is well known that the natural motions $q\left(t\right)=\{q^{i}\left(t\right)\}$ are the class of curves that make stationary the action functional

(5) $$S\left[q\left(t\right)\right]=\int_{t_{0}}^{t_{1}}L\left(\dot{q}\left(t\right),q\left(t\right)\right)dt$$

on the class of curves with $q(t_{0})=a$ and $q(t_{1})=b$ and thus isochronous paths. The Newton equations are derived from the Euler–Lagrange equations, i.e.,

(6) $$\frac{d^{2}q^{i}}{dt^{2}}=-\frac{\partial V}{\partial q_{i}}.$$

In a purely geometric framework, geodesics γ(s) are curves of a Riemannian manifold (M,g) endowed with a metric g that makes stationary the length functional between two fixed points, i.e.,

(7) $$\delta \ell \left[\gamma \right]=0\;\mathrm{with}\;\ell \left[\gamma \left(s\right)\right]=\int_{s_{0}}^{s_{1}}\sqrt{g_{ij}\frac{dq^{i}}{ds}\frac{dq^{j}}{ds}}ds\;\gamma \left(s_{0}\right)=a\;\gamma \left(s_{1}\right)=b.$$

A possible identification of natural motion with geodesics is provided by endowing a subspace of configuration space with the Jacobi metric as follows: Consider the total energy function

(8) $$H\left(\dot{q},q\right)=\left({\dot{q}}^{i}\frac{\partial L}{\partial {\dot{q}}^{i}}-L\right)=\frac{1}{2}a_{ij}\left(q\right){\dot{q}}^{i}{\dot{q}}^{j}+V\left(q\right)$$

which is a conserved quantity because $dH(\dot{q}\left(t\right),q\left(t\right))/dt=0$. Since the Lagrangian is a homogeneous function in ${\dot{q}}^{i}$, it follows that $H(\dot{q},q)=2W-L$ where

(9) $$W=\frac{1}{2}a_{ij}{\dot{q}}^{i}{\dot{q}}^{j}=H\left(\dot{q},q\right)-V\left(q\right)$$

is the kinetic energy. Considering a class of *isoenergetic* trajectories q(t;E) in configuration space, that is, trajectories with the same total energy value $H(\dot{q}\left(t;E\right),q\left(t;E\right))=E$, since the kinetic energy is non-negative, the trajectories of the system in configuration space belong to the region $M_{E}=\left\{q\in M|V(q)<E\right\}$. Moreover, for isoenergetic trajectories, the kinetic energy can be expressed as a function of the coordinates, i.e.,

(10) W(q(t;E))=E−V(q)

and thus, the action functional of Equation (5) can be rewritten in the form

(11) $$\begin{array}{cc} S_{E}\left[q\left(t;E\right)\right]=\int_{t_{0}}^{t_{1}}L\left(\dot{q}\left(t;E\right),q\left(t;E\right)\right)\;\;\;dt & =\int_{t_{0}}^{t_{1}}\left[E+2W(q(t;E))\right]\;\;\;dt\\ \; & =\left(t_{1}-t_{0}\right)E+\int_{t_{0}}^{t_{1}}2W\left(q\left(t;E\right)\right)\;\;dt. \end{array}$$

as we are interested in the variational principle and $t_{0},t_{1},E$ are fixed quantities, the first term in the last equality can be neglected. The integral in Equation (11) can be interpreted as a length integral in configuration space; in fact,

(12) $$\begin{array}{cc} S_{E}\left[q\left(t;E\right)\right] & =\int_{t_{0}}^{t_{1}}2W\left(q\left(t;E\right)\right)\;\;dt\\ \; & =\int_{t_{0}}^{t_{1}}\sqrt{2W(q(t;E))}\sqrt{2W(q(t;E))}\;\;dt=\int_{t_{0}}^{t_{1}}\sqrt{2\left[E-V(q)\right]}\sqrt{a_{ij}\left(q\right){\dot{q}}^{i}{\dot{q}}^{j}}\;\;dt=\\ \; & =\int_{t_{0}}^{t_{1}}\sqrt{2\left[E-V(q)\right]a_{ij}{\dot{q}}^{i}{\dot{q}}^{j}}\;\;dt=\int_{t_{0}}^{t_{1}}\sqrt{g_{ij}{\dot{q}}^{i}{\dot{q}}^{j}}\;\;dt=\int_{t_{0}}^{t_{1}}\sqrt{{\left(\frac{ds}{dt}\right)}^{2}}\;\;dt\\ \; & =\int_{s_{0}}^{s_{1}}ds \end{array}$$

where the new metric

(13) $$g_{ij}=2W\left(q\right)a_{ij},\;W\left(q\right)=E-V\left(q\right)$$

called the *Jacobi metric*, is introduced with the associated arc length element

(14) $$ds^{2}=g_{ij}dq^{i}dq^{j}=2\left[E-V(q)\right]a_{ij}dq^{i}dq^{j}=4{\left[W\left(q\right)\right]}^{2}dt^{2}.$$

A *conformal rescaling* of the kinetic energy metric $a_{ij}$ via a factor proportional to the kinetic energy [E−V(q)] gives the Jacobi metric, preserving the signature of the metric only inside $M_{E}=\left\{q\in M|V(q)<E\right\}$. By endowing the region $M_{E}$ with the Jacobi metric g, *the natural motions with fixed energy E are the same as the geodesics γ(s) of the manifold $\left(M_{E},g\right)$*, that is

(15) $$\frac{d^{2}q^{i}}{ds^{2}}+\Gamma_{jk}^{i}\frac{dq^{j}}{ds}\frac{dq^{k}}{ds}=0,$$

and using the definition of the Christoffel symbols $\Gamma_{jk}^{i}$

(16) $$\Gamma_{jk}^{i}=\frac{1}{2}g^{im}\left(\frac{\partial g_{km}}{\partial x^{j}}+\frac{\partial g_{mj}}{\partial x^{k}}-\frac{\partial g_{jk}}{\partial x^{m}}\right),$$

it is straightforward to show that Equation (15) becomes

(17) $$\frac{d^{2}q^{i}}{ds^{2}}+\frac{1}{2(E-V)}\left[2\frac{\partial (E-V)}{\partial q_{j}}\frac{dq^{j}}{ds}\frac{dq^{i}}{ds}-g^{ij}\frac{\partial (E-V)}{\partial q_{j}}g_{km}\frac{dq^{k}}{ds}\frac{dq^{m}}{ds}\right]=0,$$

whence, using Equation (14), Newton’s equations

(18) $$\frac{d^{2}q^{i}}{dt^{2}}=-\frac{\partial V}{\partial q_{i}}\;$$

are recovered.

### 2.2. The Eisenhart Metric in Enlarged Configuration Space

Another way of geometrizing Hamiltonian/Newtonian dynamics consists in considering as ambient space the configuration spacetime with an extra dimension—proportional to the action—$M\times R^{2}$, with local coordinates $\left(q^{0},q^{1},\ldots ,q^{i},\ldots ,q^{N},q^{N+1}\right)$. Endowed with a nondegenerate pseudo-Riemannian metric introduced by Eisenhart [17], this space has the following arc length

(19) $$ds^{2}={\left(g_{E}\right)}_{\mu \nu }\;dq^{\mu }dq^{\nu }=a_{ij}\;dq^{i}dq^{j}-2V\left(q\right){\left(dq^{0}\right)}^{2}+2\;dq^{0}dq^{N+1},$$

where μ and ν run from 0 to N+1 and i and j run from 1 to N, which is the so-called *Eisenhart metric* of metric tensor $g_{E}$. Hence, the canonical projection of the geodesics of $\left(M\times R^{2},g_{E}\right)$ on the configuration spacetime, $\pi :M\times R^{2}\mapsto M\times R$, entails the natural motions of a Hamiltonian dynamical system. Only those geodesics whose arc lengths are positive definite and given by [18]

(20) $$ds^{2}=c_{1}^{2}dt^{2}$$

correspond to natural motions; the condition (20) can be equivalently formulated in integral form for the extra coordinate $q^{N+1}$:

(21) $$q^{N+1}=\frac{c_{1}^{2}}{2}t+c_{2}^{2}-\int_{0}^{t}L\;d\tau ,$$

where $c_{1}$ and $c_{2}$ are given as real constants. Conversely, given a point P∈M×R belonging to a trajectory of the system, and given two constants $c_{1}$ and $c_{2}$, the point $P^{\prime }=\pi^{-1}\left(P\right)\in M\times R^{2}$, with $q^{N+1}$ given by (21), describes a geodesic curve in $\left(M\times R^{2},g_{E}\right)$ such that $ds^{2}=c_{1}^{2}dt^{2}$. Since the constant $c_{1}$ is arbitrary, $c_{1}^{2}=1$ can be taken so that $ds^{2}=dt^{2}$ on the physical geodesics.

From Equation (19), it follows that the explicit table of the components of the Eisenhart metric is

(22) $$g_{E}=\left(\begin{array}{ccccc} -2V(q) & 0 & \cdots & 0 & 1\\ 0 & a_{11} & \cdots & a_{1N} & 0\\ \vdots & \vdots & \ddots & \vdots & \vdots \\ 0 & a_{N1} & \cdots & a_{NN} & 0\\ 1 & 0 & \cdots & 0 & 0 \end{array}\right)$$

where $a_{ij}$ is the kinetic energy metric. The nonvanishing Christoffel symbols, in the case $a_{ij}=\delta_{ij}$, are only

(23) $$\Gamma_{00}^{i}=-\Gamma_{0i}^{N+1}=\partial_{i}V,$$

so that the geodesic Equations (15) read

(24) $$\frac{d^{2}q^{0}}{ds^{2}}=0,$$

(25) $$\frac{d^{2}q^{i}}{ds^{2}}+\Gamma_{00}^{i}\frac{dq^{0}}{ds}\frac{dq^{0}}{ds}=0,$$

(26) $$\frac{d^{2}q^{N+1}}{ds^{2}}+\Gamma_{0i}^{N+1}\frac{dq^{0}}{ds}\frac{dq^{i}}{ds}=0;$$

using ds=dt, one obtains

(27) $$\frac{d^{2}q^{0}}{dt^{2}}=0,$$

(28) $$\frac{d^{2}q^{i}}{dt^{2}}=-\frac{\partial V}{\partial q_{i}},$$

(29) $$\frac{d^{2}q^{N+1}}{dt^{2}}=-\frac{dL}{dt}.$$

Equation (27) only states that $q^{0}=t$, The N Equation (28) are Newton’s equations, and Equation (29) is the differential version of Equation (21).

### 2.3. Geodesic Spread Equation to Tackle Dynamical (In)Stability

The equation for the geodesic spread—that describes the stability of a geodesic flow of a Riemannian manifold—is the crucial mathematical tool to investigate dynamical chaos. The geodesic spread is quantified by a vector field J locally, giving the distance between nearby geodesics. This vector field evolves along a reference geodesic according to the Jacobi–Levi–Civita equation, whose components expression reads

(30) $$\frac{\nabla^{2}J^{i}}{ds^{2}}+R_{jkl}^{i}\frac{d\gamma^{j}}{ds}J^{k}\frac{d\gamma^{l}}{ds}=0$$

where

(31) $$R_{jkl}^{i}=\partial_{k}\Gamma_{jl}^{i}-\partial_{l}\Gamma_{jk}^{i}+\Gamma_{lj}^{m}\Gamma_{km}^{i}-\Gamma_{kj}^{m}\Gamma_{ml}^{i}$$

is the Riemann curvature tensor. The geodesic separation vector J is orthogonal to the velocity vector along the geodesic $\dot{\gamma }$, i.e.,

(32) $$\langle J,\dot{\gamma }\rangle =0,$$

where 〈•,•〉 represents the scalar product induced by the metric. Remarkably, the stability or instability of a reference geodesic—which is described by the evolution of J—is completely determined by the *curvature* of the manifold. Thus, if the metric is associated with a physical system, as is the case of Jacobi or Eisenhart metrics, this equation relates the stability or instability of the trajectories to the curvature of the ambient manifold.

From $\left(\nabla J^{k}/ds\right)=dJ^{k}/ds+\Gamma_{ij}^{k}\;\left(dq^{i}/ds\right)\;J^{j}$, after trivial algebra

(33) $$\frac{\nabla^{2}}{ds^{2}}J^{k}=\frac{d^{2}J^{k}}{ds^{2}}+2\Gamma_{ij}^{k}\frac{dq^{i}}{ds}\frac{dJ^{j}}{ds}+\left(\partial_{r}\Gamma_{ij}^{k}+\Gamma_{rt}^{k}\Gamma_{ij}^{t}-\Gamma_{tj}^{k}\Gamma_{ri}^{t}\right)\frac{dq^{r}}{ds}\frac{dq^{i}}{ds}J^{j},$$

where $\partial_{i}\equiv \partial /\partial q^{i}$. Then, with the above expression for the components of the Riemann–Christoffel tensor, we finally obtain [19]

(34) $$\frac{d^{2}J^{k}}{ds^{2}}+2\Gamma_{ij}^{k}\frac{dq^{i}}{ds}\frac{dJ^{j}}{ds}+\left(\frac{\partial \Gamma_{ri}^{k}}{\partial q^{j}}\right)\;\frac{dq^{r}}{ds}\frac{dq^{i}}{ds}\;J^{j}=0,$$

which has general validity *independently* of the metric of the ambient manifold.

#### 2.3.1. Curvature of the Mechanical Manifolds

The Jacobi metric is conformally flat when it is a conformal deformation of a diagonal kinetic energy metric $a_{ij}$. This greatly simplifies the computation of curvatures with the aid of a symmetric tensor C defined with components

(35) $$C_{ij}=\frac{N-2}{4{\left(E-V\right)}^{2}}\left[2\left(E-V\right)\partial_{i}\partial_{j}V+3\partial_{i}V\partial_{j}V-\frac{\delta_{ij}}{2}{\left|\nabla V\right|}^{2}\right],$$

where V is the potential, E is the energy, and ∇ and |·| stand for the Euclidean gradient and norm, respectively. The curvature of $\left(M_{E},g_{J}\right)$ can be expressed through C. In fact, the components of the Riemann tensor are

(36) $$R_{ijkm}=\frac{1}{N-2}\left[C_{jk}\delta_{im}-C_{jm}\delta_{ik}+C_{im}\delta_{jk}-C_{ik}\delta_{jm}\right].$$

By contraction of the first and third indices, we obtain the Ricci tensor, whose components are

(37) $$R_{ij}=\frac{N-2}{4{\left(E-V\right)}^{2}}\left[2\left(E-V\right)\partial_{i}\partial_{j}V+3\partial_{i}V\partial_{j}V\right]+\frac{\delta_{ij}}{4{\left(E-V\right)}^{2}}\left[2\left(E-V\right)▵V-{\left(N-4\right)|\nabla V|}^{2}\right],$$

and by a further contraction, we obtain the scalar curvature (N>2)

(38) $$R=\frac{N-1}{4{\left(E-V\right)}^{3}}\left[2\left(E-V\right)▵V-{\left(N-6\right)|\nabla V|}^{2}\right].$$

A great advantage from a computational point of view is given by the Eisenhart metric whose curvature properties are much simpler than those of the Jacobi metric. The only nonvanishing components of the Eisenhart curvature tensor are

(39) $$R_{0i0j}=\partial_{i}\partial_{j}V,$$

and hence, the Ricci tensor has only one nonzero component

(40) $$R_{00}=▵V,$$

and the scalar curvature is identically vanishing,

(41) R=0.

#### 2.3.2. The Jacobi–Levi–Civita Equation for the Jacobi Metric

The final expression for the JLC equation for $\left(M_{E},g_{J}\right)$ derived from Equation (34) is

(42) $$\begin{array}{ccc} \frac{d^{2}J^{k}}{dt^{2}} & + & \frac{1}{E-V}\left(\partial_{k}V\delta_{ij}\frac{dq^{i}}{dt}-\partial_{j}V\frac{dq^{k}}{dt}\right)\frac{dJ^{j}}{dt}+\left[\partial_{kj}^{2}V\right]\;J^{j}\\ & + & \frac{1}{E-V}\left[\left(\partial_{k}V\right)\left(\partial_{j}V\right)-\left(\partial_{ij}^{2}V+\frac{\left(\partial_{i}V\right)\left(\partial_{j}V\right)}{E-V}\right)\frac{dq^{i}}{dt}\frac{dq^{k}}{dt}\right]J^{j}=0. \end{array}$$

#### 2.3.3. The Jacobi–Levi–Civita Equation for the Eisenhart Metric

It is a very interesting fact that the JLC equation, when explicitly worked out for the Eisenhart metric on $M\times R^{2}$, yields the usual tangent dynamics equation for Hamiltonian flows. In fact,

(43) $$\begin{array}{ccc} \frac{\nabla^{2}J^{0}}{ds^{2}}+R_{i0j}^{0}\frac{dq^{i}}{ds}J^{0}\frac{dq^{j}}{ds}+R_{0ij}^{0}\frac{dq^{0}}{ds}J^{i}\frac{dq^{j}}{ds} & = & 0,\;\;\; \end{array}$$

(44) $$\begin{array}{ccc} \frac{\nabla^{2}J^{i}}{ds^{2}}+R_{0j0}^{i}{\left(\frac{dq^{0}}{ds}\right)}^{2}J^{j}+R_{00j}^{i}\frac{dq^{0}}{ds}J^{0}\frac{dq^{j}}{ds}+R_{j00}^{i}\frac{dq^{j}}{ds}J^{0}\frac{dq^{0}}{ds} & = & 0,\;\;\; \end{array}$$

(45) $$\begin{array}{ccc} \frac{\nabla^{2}J^{N+1}}{ds^{2}}+R_{i0j}^{N+1}\frac{dq^{i}}{ds}J^{0}\frac{dq^{j}}{ds}+R_{ij0}^{N+1}\frac{dq^{i}}{ds}J^{j}\frac{dq^{0}}{ds} & = & 0.\;\;\; \end{array}$$

Since $\Gamma_{ij}^{0}=0$ [see (23)], we obtain, from the definition of covariant derivative, $\nabla J^{0}/ds=dJ^{0}/ds$, and since $R_{\;ijk}^{0}=0$, we find that (43) becomes

(46) $$\frac{d^{2}J^{0}}{ds^{2}}=0,$$

such that $J^{0}$ has no acceleration, and without loss of generality, we can put ${\left.\frac{dJ^{0}}{ds}\right|}_{s=0}=J^{0}\left(0\right)=0$. This latter result combined with the definition of covariant derivative gives

(47) $$\frac{\nabla J^{i}}{ds}=\frac{dJ^{i}}{ds}+\Gamma_{0k}^{i}\frac{dq^{0}}{ds}J^{k},$$

and using $\Gamma_{0k}^{i}=0$, we obtain

(48) $$\frac{\nabla^{2}J^{i}}{ds^{2}}=\frac{d^{2}J^{i}}{ds^{2}},$$

so that after (44), the projection in configuration space of the separation vector reads

(49) $$\frac{d^{2}J^{i}}{ds^{2}}+\frac{\partial^{2}V}{\partial q_{i}\partial q^{k}}{\left(\frac{dq^{0}}{ds}\right)}^{2}J^{k}=0.$$

Equation (45) describes the evolution of the component $J^{N+1}$, which, after $g_{N+1N+1}=0$ does not contribute to the norm of J; therefore, we can disregard Equation (45).

**Figure 1:**
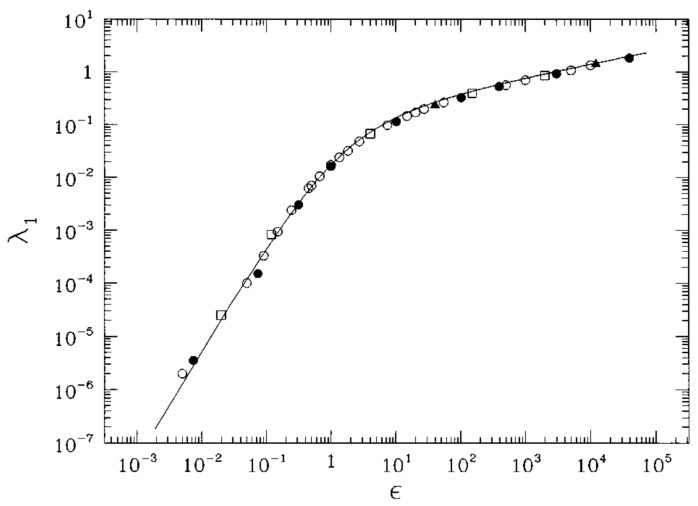
$\lambda_{1}\left(\epsilon \right)$ computed with Equation (42) at N=128 is represented by full circles ad computed at N=256 by full triangles. The largest Lyapunov exponent $\lambda_{1}\left(\epsilon \right)$ computed with Equation (50) is represented by open circles (N=256) and open squares (N=2000). The solid line is the analytic prediction for $\lambda_{1}\left(\epsilon \right)$ as discussed in the following section.

Along the physical geodesics of $g_{E}$, $ds^{2}={\left(dq^{0}\right)}^{2}=dt^{2}$ such that (49) is exactly the usual tangent dynamics equation

(50) $$\frac{d^{2}J^{i}}{dt^{2}}+\frac{\partial^{2}V}{\partial q_{i}\partial q^{k}}\;J^{k}=0.$$

that is, the usual tangent dynamics equation for Hamiltonian flows. Hence, a direct link can be made between the numerical Lyapunov exponents, the “experimental” data—so to speak—on Hamiltonian chaos, and the geometric treatment of chaotic geodesic flows. This fact is an important point in the development of a geometric theory of Hamiltonian chaos because this means that we are not introducing any new definition of chaos in the geometric context. Actually, in Figure 1, the numerical results are reported for the quantity

(51) $$\lambda_{1}=\lim_{t\to \infty }\frac{1}{t}\log\frac{{\left[J_{1}^{2}\left(t\right)+\cdots +J_{N}^{2}\left(t\right)+{\dot{J}}_{1}^{2}\left(t\right)+\cdots +{\dot{J}}_{N}^{2}\left(t\right)\right]}^{1/2}}{{\left[J_{1}^{2}\left(0\right)+\cdots +J_{N}^{2}\left(0\right)+{\dot{J}}_{1}^{2}\left(0\right)+\cdots +{\dot{J}}_{N}^{2}\left(0\right)\right]}^{1/2}},$$

computed with Equations (42) and (50), respectively, for the FPU model defined in Equation (57). And full agreement is found. Some misleading papers have appeared in the literature, casting doubts on the validity of the description of local dynamical instability based on the Jacobi metric description of Newtonian dynamics [20,21]. These claims have been disproved in Ref. [22].

### 2.4. Analytic Computation of the Largest Lyapunov Exponent

**Figure 2:**
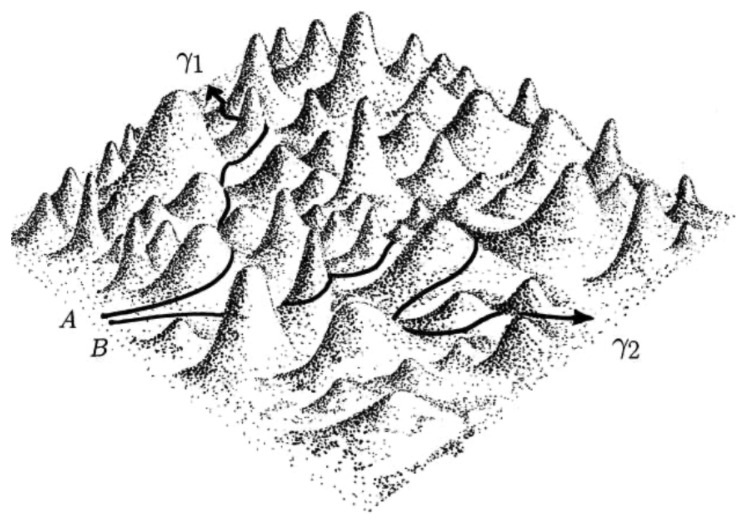
Pictorial representation of how two geodesics—$\gamma_{1}$ and $\gamma_{2}$, issuing respectively from the close points A and B—separate on a 2D “bumpy” manifold where the variations of curvature activate parametric instability.

As already mentioned above, several efforts—that followed Krylov’s pioneering work—to resort to the Riemannian geometrization of Hamiltonian dynamics to explain the origin of chaos in these dynamical systems have been unsuccessful. In fact, consider for example the Hénon–Heiles Hamiltonian system

(52) $$H=\frac{1}{2}\left(p_{x}^{2}+p_{y}^{2}\right)+\frac{1}{2}\left(x^{2}+y^{2}\right)+x^{2}y-\frac{1}{3}y^{3}.$$

where chaos was numerically detected for the first time [23], and consider the Jacobi–Levi–Civita equation, which—written for N=2 and for the Jacobi metric—exactly reads as

(53) $$\frac{d^{2}J}{ds^{2}}+\frac{1}{2}R\left(s\right)\;J=0,$$

where

(54) $$R=\frac{{\left(\nabla V\right)}^{2}}{{\left(E-V\right)}^{3}}+\frac{▵V}{{\left(E-V\right)}^{2}},$$

is the scalar curvature of the manifold $\left(M_{E},g_{J}\right)$ which, after Equation (52), turns out to be always positive and therefore at odds with the widespread expectation of geodesic instability and thus chaos, stemming from negatively curved manifolds. The idea that chaos in Newton dynamics has to be associated with hyperbolicity followed some important theoretical results [24,25,26,27,28] that gave rise to abstract ergodic theory. The turning point for the successful use of the Riemannnian geometrization to explain the origin of chaos in Hamiltonian flows was the numerical investigation [14] of the curvature properties probed by the geodesics representing the trajectories of a Hamiltonian system, which showed that for flows of physical interest, it is the variations in curvature rather than the hyperbolicity of the underlying manifold that destabilize the geodesics/trajectories (see Figure 2 for a pictorial illustration of this mechanism). The variability in the curvature probed by a geodesic activates *parametric instability* [29] even on all positively curved manifolds. In particular, for the Hénon–Heiles model, this has been studied in detail in Ref. [30]. The geometrization of dynamics provides a unique fundamental explanation of the origin of chaos, and, at least in some computable cases, it allows to get rid of the numerical computation of the Lyapunov exponent along with the numerical computation of the dynamics. We refer the reader to see elsewhere [14,15,16,31] for the details of a successful strategy to work out the following effective instability equation:

(55) $$\frac{d^{2}\psi }{ds^{2}}+{\langle k_{R}\rangle }_{\mu }\;\psi +{\langle \delta^{2}k_{R}\rangle }_{\mu }^{1/2}\;\eta \left(s\right)\;\psi =0,$$

where ψ is such that $∥\psi^{2}\left(t\right)∥∼∥J^{2}\left(t\right)∥$, $k_{R}$ is the Ricci curvature of the mechanical manifold, ${\langle \cdot \rangle }_{\mu }$ stands for averaging on it, and η(s) is a Gaussian-distributed δ-correlated random process. This equation is *independent of the dynamics* and depends only on some total curvature property of the mechanical manifold. Equation (55) has the form of a stochastic oscillator equation whose solution has an exponentially growing envelope due to parametric instability, which seems to be a ubiquitous mechanism responsible for chaos in physical Hamiltonians. The geometrization of dynamics allows the development of a “statistical–mechanical” description of the average strength of chaos, bringing about the possibility of an analytic computation of the largest Lyapunov exponent through the general formula (56) for the rate of the exponential growth of $∥\psi^{2}\left(t\right)∥+∥{\dot{\psi }}^{2}\left(t\right)∥$, which is worked out by means of van Kampen’s method [32] to give the following [15,16,31].

(56) $$\begin{array}{ccc} \lambda (k_{0},\sigma_{k},\tau ) & = & \frac{1}{2}\left(\Lambda -\frac{4k_{0}}{3\Lambda }\right),\\ \Lambda & = & {\left(\sigma_{k}^{2}\tau +\sqrt{{\left(\frac{4k_{0}}{3}\right)}^{3}+\sigma_{k}^{4}\tau^{2}}\;\right)}^{1/3}, \end{array}$$

where $k_{0}={\langle k_{R}\rangle }_{\mu }$, $\sigma_{k}={\langle \delta^{2}k_{R}\rangle }_{\mu }$ and τ is a characteristic time defined through a geometric argument. The two following “paradigmatic” applications have proven the validity and power of the geometric approach. The first is in the case of a chain of harmonic oscillators also coupled through a quartic anharmonic potential (the FPU β-model) described by the Hamiltonian

(57) $$H\left(p,q\right)=\sum_{k=1}^{N}\left[\frac{1}{2}p_{k}^{2}+\frac{1}{2}{\left(q_{k+1}-q_{k}\right)}^{2}+\frac{\beta }{4}{\left(q_{k+1}-q_{k}\right)}^{4}\right]$$

which in normal modes (phonons) also reads as

(58) $$H\left(P,Q\right)=\frac{1}{2}\sum_{k=1}^{N}\left(P_{k}^{2}+\omega_{k}^{2}Q_{k}^{2}\right)+\beta \sum_{i,j,k,l=1}^{N}D_{ijkl}Q_{i}Q_{j}Q_{k}Q_{l},$$

and the averages $k_{0}$$\sigma_{k}$ can be analytically computed using the Eisenhart metric so that after Equation (56), the impressive result of Figure 3 is found.

**Figure 4:**
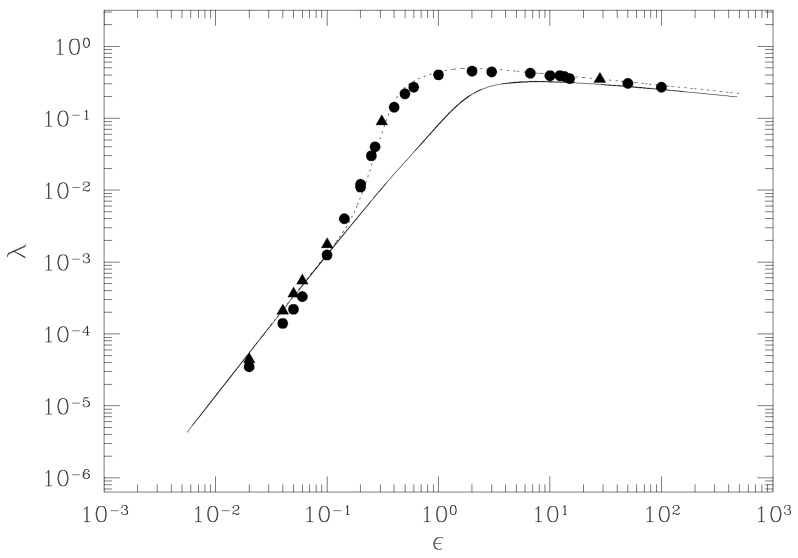
Lyapunov exponent λ vs. energy density ϵ for the 1d-XY model with J=1. The continuous line is the theoretical computation according to (56), while full circles, squares, and triangles are the results of numerical simulations with N, respectively, equal to 150, 1000, and 1500. The dotted line is the theoretical result, where the value of $k_{0}$ has been corrected to account for an excess of negative values of $k_{R}$ in an intermediate interval of ϵ values.

Second, in the case of a chain of coupled rotators, also referred to as the 1d-XY classical spin model described by

(59) $$H=\sum_{i=1}^{N}\frac{1}{2}{\dot{s}}_{i}^{2}+J\sum_{i=1}^{N}s_{i+1}\cdot s_{i}$$

that, for unimodular spin variables ($|s_{i}|=1$), reads as

(60) $$H\left(p,q\right)=\sum_{i=1}^{N}\left\{\frac{p_{i}^{2}}{2}+J\left[1-\cos\left(\theta_{i+1}-\theta_{i}\right)\right]\right\},$$

it is again possible to analytically compute the averages $k_{0}$$\sigma_{k}$ using the Eisenhart metric so that after Equation (56), another impressive result given in Figure 4 is found (in this case, a correction to the distribution of $k_{R}$ is necessary: see Ref. [31]).

## 3. Geometry and Chaos at Phase Transitions

As already mentioned in the Introduction, the macroscopic properties of large-N Hamiltonian systems can be understood using traditional statistical mechanics methods. Among the others, there is a class of phenomena where the macroscopic thermodynamic quantities can change dramatically when an external control parameter (e.g., temperature or energy) crosses a critical value. These are collective phenomena called phase transitions empirically identified by discontinuities of macroscopic thermodynamic quantities. From a theoretical viewpoint, phase transitions are explained as true mathematical singularities that occur in thermodynamic functions in the thermodynamic limit, that is, N→∞. Now, the main hypothesis at the grounds of statistical mechanics is ergodicity of the underlying dynamics, a property which is bona fide ensured by dynamical chaoticity. It is therefore natural to wonder whether there is any specific signature provided by the largest Lyapunov exponent when a system undergoes a phase transition, and, if this is the case, what the geometric changes of the configuration-space manifold are in the presence of a phase transition, in view of the above-described geometric description of chaos.

Let us consider the XY model in three spatial dimensions described by the Hamiltonian

(61) $$\begin{array}{ccc} H=\sum_{i,j,k=1}^{n}\{\frac{1}{2}p_{i,j,k}^{2}+J[ & 3 & -\cos\left(q_{i+1,j,k}-q_{i,j,k}\right)-\cos\left(q_{i,j+1,k}-q_{i,j,k}\right)\\ & - & \cos(q_{i,j,k+1}-q_{i,j,k})]\}. \end{array}$$

**Figure 5:**
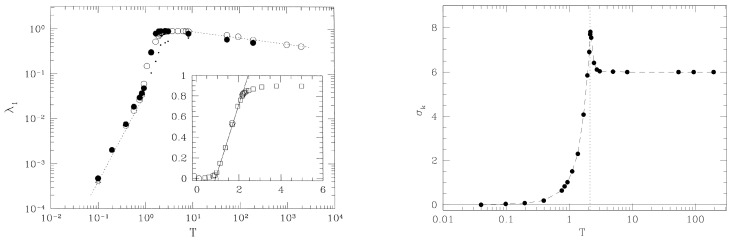
**Left panel**: Lyapunov exponent λ vs. the temperature T for the three-dimensional XY model, defined in (61), numerically computed on an N=10×10×10 lattice (solid circles) and on an N=15×15×15 lattice (solid squares). The critical temperature of the phase transition is $T_{c}\approx 2.15$. From [7]. **Right panel**: Fluctuations of the Ricci curvature (Eisenhart metric), for the same model. Here, N=10×10×10. The critical temperature of the phase transition is marked by a vertical dotted line.

**Figure 6:**
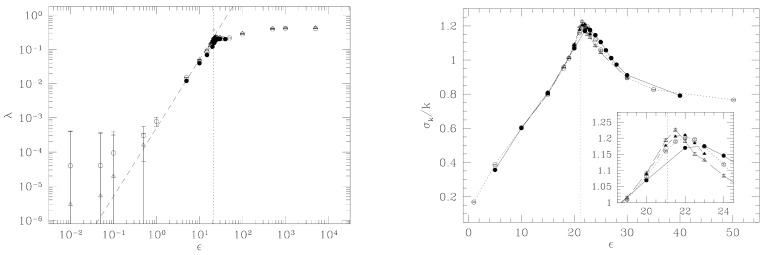
**Left panel**: Lyapunov exponent λ vs. the energy per particle ε, numerically computed for the two-dimensional $Z_{2}$$\varphi^{4}$ model, with N=100 (solid circles), N=400 (open circles), N=900 (solid triangles), and N=2500 (open triangles). The critical energy is marked by a vertical dotted line, and the dashed line is the power law $\varepsilon^{2}$. From [9]. **Right panel**: Root mean square fluctuation of the Ricci curvature (Eisenhart metric) $\sigma_{k}$, divided by the average curvature $k_{0}$, numerically computed for the same model. The inset shows a magnification of the region close to the transition.

This model undergoes a standard continuous (second-order) phase transition accompanied by the breaking of the O(2) symmetry of the Hamiltonian. The behavior of the largest Lyapunov exponent $\lambda_{1}$ as a function of the temperature T is shown in Figure 5, where the appearance of an “angular” point is evident in the pattern of $\lambda_{1}\left(T\right)$ at the transition point, which is even more evident in the inset of Figure 5. The pattern $\lambda_{1}\left(\epsilon \right)$ reported in Figure 4 for a system having the same symmetry—but in the absence of phase transitions—is definitely smoother than the one of Figure 5 (despite the difference in the variable in abscissas). Another clear example of how the energy pattern of the largest Lyapunov exponent looks very different in the presence versus in the absence of a phase transition is provided by the comparison between $\lambda_{1}\left(\epsilon \right)$ reported in Figure 3 versus the one in Figure 6, the latter referring to the so-called lattice $\varphi^{4}$ model, i.e., a system described by the Hamiltonian

(62) $$H\left(p,q\right)=\sum_{i\in Z^{d}}\left(\frac{\pi_{i}^{2}}{2}-\frac{\mu^{2}}{2}\varphi_{i}^{2}+\frac{\lambda }{4!}\varphi_{i}^{4}\right)+\sum_{\langle \mathrm{ik}\rangle \in Z^{d}}\frac{1}{2}J{\left(\varphi_{i}-\varphi_{k}\right)}^{2},$$

where $\varphi_{i}\in \left(-\infty ,+\infty \right)$ are scalar variables defined on the sites of a d-dimensional lattice, $\pi_{i}$ are the conjugated momenta, and 〈ik〉 stands for nearest-neighbor sites on the lattice. This system has a discrete $Z_{2}$-symmetry and short-range interactions; therefore, according to the Mermin–Wagner theorem, in d=1, there is no phase transition, whereas in d=2, there is a second-order symmetry-breaking transition, with nonzero critical temperature, of the same universality class of the 2d Ising model.

Also, the Hamiltonian of the FPU β-model is invariant under $Z_{2}$ transformations, but being one-dimensional, it does not undergo a phase transition. A sharp “cuspy” point of $\lambda_{1}\left(\epsilon \right)$ is observed at the critical energy value of the phase transition of the lattice $\varphi^{4}$ model. To the contrary, the pattern of $\lambda_{1}\left(\epsilon \right)$ for the FPU model is definitely smooth. Since Lyapunov exponents are tightly related to the geometry of the mechanical submanifolds of configuration space, it is natural to wonder what happens to the geometry of these manifolds in correspondence of the “cuspy” energy—or temperature—patterns of $\lambda_{1}$. The geometric quantities that enter the analytic Formula (56) for $\lambda_{1}$ are the average Ricci curvature and its variance. The interesting result is that the phase transition point is marked by a peak in the curvature fluctuations as is shown in the right panels of Figure 5 and Figure 6. This fact is invariably observed for all the models undergoing phase transitions that are studied also from this geometrical viewpoint. The observation that the topology change driven by a continuously varying parameter in a family of two dimensional–surfaces is accompanied by a sharp peak in the variance of the Gaussian curvature suggests that the observed geometrical signature of phase transitions hints at the possible relevant role of topology [16].

### Lyapunov Exponents and Configuration Space Topology

**Figure 7:**
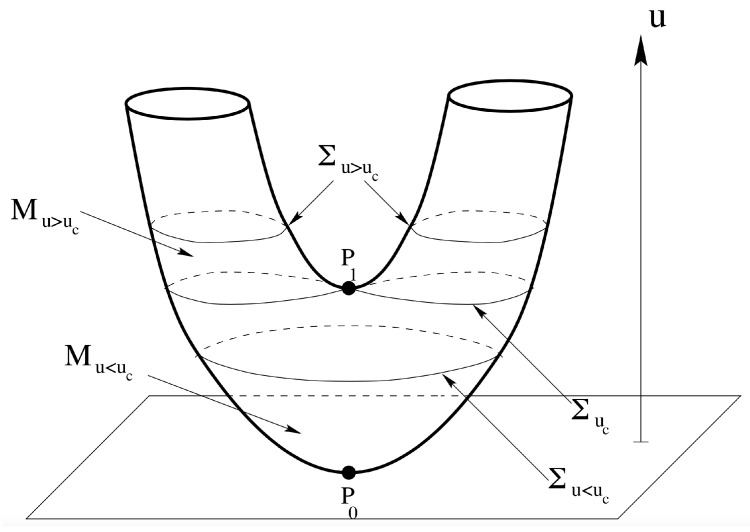
Here, the function f is the height of a point of the bent cylinder with respect to the ground. In $P_{1}$, it is df=0. The level sets $\Sigma_{u}=f^{-1}\left(u\right)$ below this critical point are circles, whereas above are the union of two circles. The manifolds $M_{u}=f^{-1}\left(\left(-\infty ,u\right]\right)$ are disks for $u<u_{c}$ and cylinders for $u>u_{c}$.

Let us give an intuitive idea of the relationship between critical points of a function in a given space and the topology of its level sets by considering a low-dimensional and elementary case. Given a smooth function f, bounded below, such that $f:R^{N}\to R$, its level sets $\Sigma_{u}=f^{-1}\left(u\right)$ are diffeomorphically transformed one into the other by the flow [33]

$$\frac{dx}{du}=\frac{\nabla f}{{∥\nabla f∥}^{2}},$$

where $x\in R^{N}$, i.e., the points of a hypersurface $\Sigma_{u_{0}}$ with $u_{0}\in \left[a,b\right]\subset R$ are mapped by this flow to the points of another $\Sigma_{u_{1}}$ with $u_{1}\in \left[a,b\right]$, provided that ∇f never vanishes in the interval [a,b]. In other words, if in the interval [a,b] the function f has no critical points, all the level sets $\Sigma_{u}=f^{-1}\left(u\right)$, with u∈[a,b], have the same topology. Conversely, the appearance of critical points of f at some critical value $u_{c}$ breaks the diffeomorphicity among the $\Sigma_{u<u_{c}}$ and $\Sigma_{u>u_{c}}$. This is illustrated by one of the simplest possible examples [34] in Figure 7. A systematic study is developed within *Morse theory* of the relationship between the topological properties of a manifold and the critical points of a suitable class of real-valued functions (Morse functions) defined on it. Any Morse function can be parametrized, in the neighborhood of a critical point located at $x_{0}$, by means of the so-called Morse chart, i.e., a system of local coordinates $\left\{y_{i}\right\}$, such that $f\left(y\right)=f\left(x_{0}\right)-\sum_{i=1}^{k}y_{i}^{2}+\sum_{i=k+1}^{N}y_{i}^{2}$ (k is the Morse index of the critical point) [35]. In particular, if f≡V, Morse theory tells us that if there are critical points of V in configuration space, that is, points $q_{c}=\left[{\bar{q}}_{1},\cdots ,{\bar{q}}_{N}\right]$ such that ${\left.\nabla V(q)\right|}_{q=q_{c}}=0$, in the neighborhood of any critical point $q_{c}$, there always exists a coordinate system $\tilde{q}\left(t\right)=\left[{\tilde{q}}_{1}\left(t\right),\cdots ,{\tilde{q}}_{N}\left(t\right)\right]$ for which

(63) $$V\left(\tilde{q}\right)=V\left(q_{c}\right)-{\tilde{q}}_{1}^{2}-\cdots -{\tilde{q}}_{k}^{2}+{\tilde{q}}_{k+1}^{2}+\cdots +{\tilde{q}}_{N}^{2},$$

where the index k of the critical point is the number of negative eigenvalues of the Hessian of V computed at $q_{c}$. In the neighborhood of a critical point, Equation (63) yields $\partial_{ij}^{2}V=\pm \delta_{ij}$, which, substituted into Equation (50), gives

$$\frac{d^{2}J}{dt^{2}}+\left(\begin{array}{ccccc} -1 & 0 & \cdots & 0 & 0\\ 0 & -1 & \cdots & 0 & 0\\ \vdots & \vdots & \ddots & \vdots & \vdots \\ 0 & 0 & \cdots & 1 & 0\\ 0 & 0 & \cdots & 0 & 1 \end{array}\right)J\;=0\;,$$

where there are k unstable directions that contribute to the exponential growth of the norm of the tangent vector J. This means that the strength of dynamical chaos, measured by the largest Lyapunov exponent $\lambda_{1}$, is affected by the existence of critical points of V. Therefore, the presence of a sudden variation, as a function of the potential energy v, of the number of critical points (or of their indexes) in configuration space at some value $v_{c}$ can affect the pattern of $\lambda_{1}\left(v\right)$—as well as that of $\lambda_{1}\left(E\right)$—since v=v(E). In other words, peculiar energy patterns of $\lambda_{1}\left(E\right)$ displaying “singular” patterns—for example, jumps or cusps—at a phase transition point are suggestive of being rooted in some kind of topological change in the hypersurfaces ${\left\{\Sigma_{v}\right\}}_{v\in R}=\left\{V_{N}\left(q_{1},\cdots ,q_{N}\right)=v\in R\right\}$, and also in the ${\left\{M_{v}\right\}}_{v\in R}={\left\{V_{N}^{-1}\left(\left(-\infty ,v\right]\right)\right\}}_{v\in R}$ bounded by the $\Sigma_{v}$.

## 4. Topology and Phase Transitions

In addition to the arguments—reported in the preceding section—suggesting a possible role of topology at the roots of phase transitions, there is another reason supporting this hypothesis. This is due to a quantitative connection between the geometry and topology of the energy landscape in phase space, or in configuration space, and thermodynamic entropy defined as

(64) $$S_{N}\left(E\right)=\left(k_{B}/N\right)\log\int_{\Sigma_{E}}\;d\sigma \;/∥\nabla H∥$$

that is [16]

(65) $$S\left(E\right)=\frac{k_{B}}{N}\log\left[\mathrm{vol}\left(S_{1}^{N-1}\right)\sum_{i=0}^{N}b_{i}\left(\Sigma_{E}\right)+\int_{\Sigma_{E}}d\sigma \frac{R(E)}{2^{N}N!}\right]+r\left(E\right)\;,$$

where $b_{i}\left(\Sigma_{E}\right)$ are the Betti numbers of the constant energy hypersurfaces in phase space, and r(E) is a remainder function. Betti numbers are fundamental topological invariants under diffeomorphisms of a manifold [36]. Another version of this formula reads

(66) $$S\left(v\right)=\frac{k_{B}}{N}\log\left[\mathrm{vol}\left(S_{1}^{N-1}\right)\left(\mu_{0}+\sum_{i=1}^{N-1}2\mu_{i}\left(M_{v}\right)+\mu_{N}\right)+\tilde{R}\left(E\left(v\right)\right)\right]+\tilde{r}\left(E\left(v\right)\right),$$

which now holds in configuration space and where the $\mu_{i}\left(M_{v}\right)$ are the Morse indexes (in one-to-one correspondence with topology changes) of the submanifolds $M_{v}$ of configuration space. These formulas are approximate, but, following a different conceptual path and using the definition $S_{N}^{\left(-\right)}\left(v\right)=\left(k_{B}/N\right)\log\int_{M_{v}}\;d^{N}q$, an exact formula can also be derived, which reads [16]

(67) $$S_{N}^{\left(-\right)}\left(v\right)=\frac{k_{B}}{N}\log\left[\mathrm{vol}\left[M_{v}\setminus \overset{N(v)}{\underset{i=1}{⋃}}\Gamma \left(x_{c}^{\left(i\right)}\right)\right]\;+\sum_{i=0}^{N}w_{i}\;\mu_{i}\left(M_{v}\right)+R\left(N,v\right)\right],$$

where the first term in the square brackets is the configuration-space volume minus the sum of volumes of certain neighborhoods of the critical points of the interaction potential; the second term is a weighed sum of the Morse indexes $\mu_{k}$ ($\mu_{k}$ is number of critical points of index k); and the third term is a smooth function. The above formula is of special interest because it provides an exact relation between entropy and some quantities of topological meaning. It is evident that abrupt changes in the potential energy pattern of at least some of the $\mu_{i}\left(M_{v}\right)$ (thus, of the way topology changes with v) affect $S_{N}\left(v\right)$ and its derivatives; equivalently, abrupt changes in the total energy pattern of the $b_{k}\left(\Sigma_{E}\right)$ (thus, of the way topology changes with E) affect S(E) and its derivatives. All the arguments given hitherto lead to the formulation of a *topological hypothesis*. Concisely, consider the microcanonical volume

(68) $$\Omega \left(E\right)=\int_{0}^{E}d\eta \;\frac{{\left(2\pi \eta \right)}^{N/2}}{\eta \Gamma (\frac{N}{2})}\int_{0}^{E-\eta }du\;\int_{\Sigma_{u}}\frac{d\sigma }{∥\nabla V∥};$$

where the larger that N is, the closer to some $\Sigma_{u}$ the microscopic configurations are that significantly contribute to the statistical averages, and therefore, the idea is that in order to observe the development of singular behaviors of thermodynamic observables at some critical value $u_{c}$, it is *necessary* to break the diffeomorphicity between the families ${\left\{\Sigma_{u}\right\}}_{u<u_{c}}$ and ${\left\{\Sigma_{u}\right\}}_{u>u_{c}}$, that is, the $\Sigma_{u}$ need to undergo a topology change at $u_{c}$.

### 4.1. Rigorous Results

There are two Hamiltonian systems for which we can exactly compute the partition function, and thus prove the existence of a phase transition, its order, and the transition critical values of the energy, potential energy, and temperature; moreover, for the same systems, it is possible to analytically compute all the critical points of the potential function and their Morse indexes, and hence the potential energy dependence of the Euler characteristic, a topological invariant. The rigorous outcomes confirm, at least for the special case of these two models, the topological hypothesis. In Refs. [37,38], the *topological hypothesis*, exactly confirmed for the two mentioned systems, is turned into a theorem where a *necessary* topological condition for the occurrence of first- or second-order phase transitions is established.

#### 4.1.1. Two Exactly Computable Models

The two Hamiltonian systems previously mentioned are the so-called mean-field XY model and k-trigonometric model (*k*TM).

The mean-field XY model is defined by the Hamiltonian

(69) $$H\left(p,\varphi \right)=\sum_{i=1}^{N}\frac{p_{i}^{2}}{2}+\frac{J}{2N}\sum_{i,j=1}^{N}\left[1-\cos(\varphi_{i}-\varphi_{j})\right]-h\sum_{i=1}^{N}\cos\varphi_{i}.$$

Here, $\varphi_{i}\in \left[0,2\pi \right]$ is the rotation angle of the *i*th unimodular classical spin, and h is an external field. Defining at each site i a spin vector $m_{i}=\left(\cos\varphi_{i},\sin\varphi_{i}\right)$, the model describes a planar (XY) Heisenberg system with interactions of equal strength among all the spins. The Euler characteristic can be computed analytically as a function of the potential energy per degree of freedom v according to the formula

(70) $$\chi \left(M_{v}\right)=\sum_{k=0}^{N}{\left(-1\right)}^{k}\mu_{k}\left(M_{v}\right),$$

where the *Morse number* $\mu_{k}$ is the number of critical points of the potential function in Equation (69) that have index k [34].

**Figure 8:**
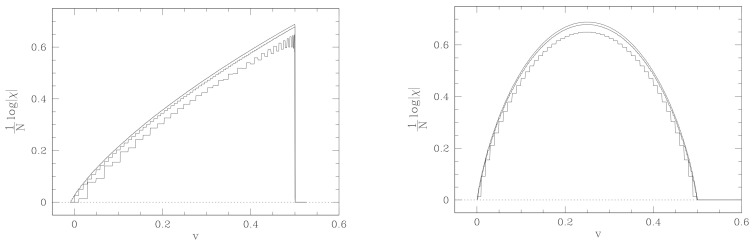
**Left panel**: Mean-field XY model. Plot of $\log(|\chi |\left(M_{v}\right))/N$ as a function of v. N= 50, 200, 800 (from bottom to top) and h=0.01; $v_{c}=0.5+O\left(h^{2}\right)$. **Right panel**: Plot of $\log(|\chi |\left(M_{v}\right))/N$ for the one-dimensional XY model with nearest-neighbor interactions as a function of v. N= 50, 200, 800 (from bottom to top).

Figure 8 shows that a sharp jump in $\chi (M_{v})$ takes place at $v_{c}=0.5$, where the system undergoes a second-order phase transition. No jump is observed in the case of the 1d-XY model having the same O(2) symmetry and spatial dimensionality of the mean-field XY model but with short-range interactions such that, after the Mermin–Wagner theorem, it does not undergo any phase transition.

**Figure 9:**
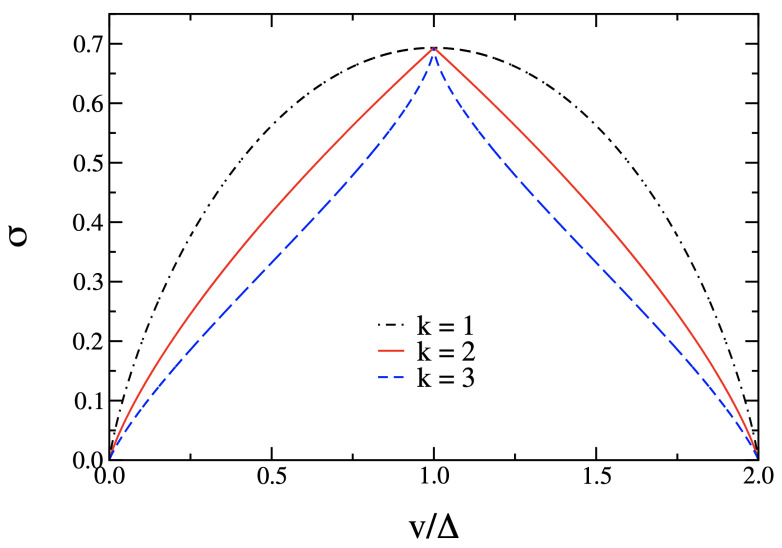
Logarithmic Euler characteristic σ(v) of the $M_{v}$ manifolds as a function of the potential energy v. The phase transition is signaled as a singularity of the first derivative at $v_{c}=\Delta$; the sign of the second derivative around the singular point allows to predict the order of the transition.

The *k*TM is defined by the Hamiltonian

(71) $$H_{k}=\sum_{j=1}^{N}\frac{1}{2}p_{j}^{2}+V_{k}\left(\varphi_{1},\ldots ,\varphi_{N}\right)\;,$$

where $\left\{\varphi_{j}\right\}$ are angular variables, $\varphi_{j}\in \left[0,2\pi \right)$ and $\left\{p_{j}\right\}$ are the conjugated momenta, and the potential energy V is given by

(72) $$V_{k}=\frac{\Delta }{N^{k-1}}\sum_{j_{1},...,j_{k}}\left[1-\cos\left(\varphi_{j_{1}}+...+\varphi_{j_{k}}\right)\right]\;,$$

where Δ is a coupling constant. Again, as in the case of the mean-field XY model, only the potential energy part is considered. This interaction energy is apparently of a mean-field nature, in that each degree of freedom interacts with all the others; moreover, the interactions are k-body ones. For k=1, no phase transition is present; for k=2, the system undergoes a second-order phase transition; and for k≥3, the system undergoes a first-order phase transition. In all these cases, the partition function can be exactly computed, and the Euler characteristic can be exactly computed as well. Figure 9 shows a clear-cut topological signature of the presence of a phase transition and of its order.

#### 4.1.2. A Necessity Theorem

The theorem establishing the necessary topological origin of a phase transition, in its original formulation, given in Refs. [37,38], was lacking a fundamental hypothesis, which led to the paradoxical situation of it being falsified [39] through the example of phase transition still linked to a change in topology in the configuration space, despite being asymptotic in the number of degrees of freedom [40], and in the absence of critical points of the potential.

The missing hypothesis suggested by the study of Ref. [40] consists in also requiring the asymptotic diffeomorphicity of the equipotential hypersurfaces to correspondingly obtain a uniform convergence of the Helmholtz free energy in a class of differentiability that excludes phase transitions of the first and second orders.

**Theorem 1**  (Absence of phase transitions under diffeomorphicity)**.** *Let $V_{N}\left(q_{1},\cdots ,q_{N}\right):R^{N}\to R$ be a smooth, nonsingular, finite-range potential. Denote by $\Sigma_{v}^{V_{N}}:=V_{N}^{-1}\left(v\right)$, v∈R, its* level sets, *or* equipotential hypersurfaces, *in configuration space.*

*Then, let $\bar{v}=v/N$ be the potential energy per degree of freedom.*

*If, for any pair of values $\bar{v}$ and ${\bar{v}}^{\prime }$ belonging to a given interval $I_{\bar{v}}=\left[{\bar{v}}_{0},{\bar{v}}_{1}\right]$ and for any $N>N_{0}$ with $N\in N^{\#}$ (that is including N→∞) we have*

$$\Sigma_{N\bar{v}}^{V_{N}}\approx \Sigma_{N{\bar{v}}^{\prime }}^{V_{N}},$$

*that is, $\Sigma_{N\bar{v}}^{V_{N}}$ is* diffeomorphic *to $\Sigma_{N{\bar{v}}^{\prime }}^{V_{N}}$, including* asymptotically diffeomorphic, *then the sequence of the Helmholtz free energies ${\left\{F_{N}\left(\beta \right)\right\}}_{N\in N}$—where β=1/T (T is the temperature) and $\beta \in I_{\beta }=\left(\beta \left({\bar{v}}_{0}\right),\beta \left({\bar{v}}_{1}\right)\right)$—is* uniformly *convergent at least in $C^{2}\left(I_{\beta }\subset R\right)$ such that $F_{\infty }\in C^{2}\left(I_{\beta }\subset R\right)$ and neither first- nor second-order phase transitions can occur in the (inverse) temperature interval $\left(\beta \left({\bar{v}}_{0}\right),\beta \left({\bar{v}}_{1}\right)\right)$.*

**Remark 1.**  *The configurational entropy $S_{N}\left(\bar{v}\right)$ is related to the configurational canonical free energy $f_{N}$ in (74) for any N∈N, $\bar{v}\in R$, and β∈R through the Legendre transform*

(73) $$\begin{array}{c} -f_{N}\left(\beta \right)=\beta \cdot {\bar{v}}_{N}-S_{N}\left({\bar{v}}_{N}\right) \end{array}$$

*where the inverse of the configurational temperature T(v) is given by $\beta_{N}\left(\bar{v}\right)=\partial S_{N}\left(\bar{v}\right)/\partial \bar{v}$. By following Ref. [41], let us consider the function $\phi \left(\bar{v}\right)=f_{N}\left[\beta \left(\bar{v}\right)\right]$, and from $\phi^{\prime }\left(\bar{v}\right)=-\bar{v}\;\left[d\beta_{N}\left(\bar{v}\right)/d\bar{v}\right]$ it is evident that if $\beta_{N}\left(\bar{v}\right)\in C^{k}\left(R\right)$, then also $\phi \left(\bar{v}\right)\in C^{k}\left(R\right)$ and thus $S_{N}\left(\bar{v}\right)\in C^{k+1}\left(R\right)$, while $f_{N}\left(\beta \right)\in C^{k}\left(R\right)$. First- and second-order phase transitions are associated with a discontinuity in the first or second derivatives of $f_{\infty }\left(\beta \right)$, that is, with $f_{\infty }\left(\beta \right)\in C^{0}\left(R\right)$ or $f_{\infty }\left(\beta \right)\in C^{1}\left(R\right)$, respectively. Hence, a first-order phase transition corresponds to a discontinuity of the second derivative of the entropy $S_{\infty }\left(\bar{v}\right)$, and a second-order phase transition corresponds to a discontinuity of the third derivative of the entropy $S_{\infty }\left(\bar{v}\right)$.*

**Remark 2.**  *The proof of the main theorem follows the same conceptual path given in Refs. [37,38]: a topological change in the equipotential hypersurfaces $\Sigma_{v}^{V_{N}}$ of the configuration space is a necessary condition for the occurrence of a thermodynamic phase transition if we prove the equivalent proposition that if any two hypersurfaces $\Sigma_{v}^{V_{N}}$ and ${\Sigma_{v^{\prime }}}^{V_{N}}$ with $v\left(N\right),v^{\prime }\left(N\right)\in \left(v_{0}\left(N\right),v_{1}\left(N\right)\right)$ are diffeomorphic for all $N\in N^{\#}$, then no phase transition can occur in the (inverse) temperature interval $\left[\lim_{N\to \infty }\beta \left({\bar{v}}_{0}\left(N\right)\right),\lim_{N\to \infty }\beta \left({\bar{v}}_{1}\left(N\right)\right)\right]$.*

For standard Hamiltonian systems (i.e., quadratic in the momenta), the relevant information is carried by the configurational microcanonical ensemble, where the configurational canonical free energy is

(74) $$f_{N}\left(\beta \right)\equiv f_{N}\left(\beta ;V_{N}\right)=\frac{1}{N}\log\int_{{\left(\Lambda^{d}\right)}^{\times n}}dq_{1}\cdots dq_{N}\;\exp\left[-\beta V_{N}\left(q_{1},\cdots ,q_{N}\right)\right]$$

and the configurational microcanonical entropy (in units s.t. $k_{B}=1$) is

$$S_{N}\left(\bar{v}\right)\equiv S_{N}\left(\bar{v};V_{N}\right)=\frac{1}{N}\log\int_{{\left(\Lambda^{d}\right)}^{\times n}}dq_{1}\cdots dq_{N}\;\delta \left[V_{N}\left(q_{1},\cdots ,q_{N}\right)-v\right]\;\;.$$

Then, $S_{N}\left(\bar{v}\right)$ is related to the configurational canonical free energy $f_{N}$ for any N∈N, $\bar{v}\in R$, and β∈R through the Legendre transform in Equation (73).

With the aid of several Lemmas [42], it is shown that in the limit N→∞ and at constant particle density $\mathrm{vol}{\left(\Lambda^{d}\right)}^{\times n}/N\;=\;\mathrm{const}$, in the interval $I_{\bar{v}}=\left[{\bar{v}}_{0},{\bar{v}}_{1}\right]$, the sequence ${\left\{S_{N}\right\}}_{N\in N^{\#}}$ is uniformly convergent in $C^{3}\left(I_{\bar{v}}\subset R\right)$ so that $S_{\infty }\in C^{3}\left(I_{\bar{v}}\subset R\right)$, that is, the thermodynamic limit of the entropy is three times differentiable, with a continuous third-order derivative, in $I_{\bar{v}}=\left[{\bar{v}}_{0},{\bar{v}}_{1}\right]$. Hence, in the interval $I_{\beta }=\left[\lim_{N\to \infty }\beta \left({\bar{v}}_{0}\left(N\right)\right),\lim_{N\to \infty }\beta \left({\bar{v}}_{1}\left(N\right)\right)\right]$, the sequence of configurational free energies ${\left\{f_{N}\left(T\right)\right\}}_{N\in N^{\#}}$ is *uniformly convergent* at least in $C^{2}\left(I_{\beta }\subset R\right)$ so that we have

$$-f_{\infty }\left(\beta \right)=\beta \left(\bar{v}\right)\cdot \bar{v}-S_{\infty }\left(\bar{v}\right)$$

that is, $\left\{f_{\infty }\left(T\right)\right\}\in C^{2}\left(I_{\beta }\subset R\right)$.

Since a quadratic kinetic energy term of a standard Hamiltonian gives only a smooth contribution to the total Helmholtz free energy $F_{N}\left(\beta \right)$, also the asymptotic function $F_{\infty }\left(\beta \right)$ has differentiability class $C^{2}\left(I_{\beta }\subset R\right)$ so that we conclude that the corresponding physical system does not undergo neither first- nor second-order phase transitions in the inverse-temperature interval $\beta \in I_{\beta }$.

This theorem is used to prove that in (67), the origin of the possible unbound growth with N of some derivative of the entropy, that is, of the development of an analytic singularity in the limit N→∞ and thus of a phase transition, can be due only to the topological term $\sum w_{i}\;\mu_{i}\left(M_{v}\right)$ [43].

It is important to remark that a topological change in the submanifolds $\Sigma_{v}$ and $M_{v}$ of the configuration space is a *necessary* but not *sufficient* condition for the occurrence of a phase transition. In fact, in Figure 8 and Figure 9, it is evident that $\chi (M_{v})$ is almost continuously changing with v, meaning that the topology of the $M_{v}$ undergoes almost continuous changes with v. It is rather the way of changing of the topology which is associated with a phase transition. Some first steps toward sufficiency conditions are given in Ref. [44].

### 4.2. Applications

In general, the computation of any topological invariant through the computation of all the critical points of the potential function of a given system is prohibitive and unfeasible, both analytically and numerically. Therefore, one has to resort to an alternative approach in order to obtain information on the topology of the manifolds of interest. This can be performed by resorting to theorems in differential topology relating the total geometric quantities of a given manifold with its topology. With “total”, it is meant the integral of a given quantity over the whole manifold. The following theorems can be applied in the framework of numerical computations.

The first important result is the Gauss–Bonnet–Hopf theorem [45,46], which is expressed by the following equation:

(75) $$\int_{\Sigma_{v}}d\sigma \;K_{G}=\frac{1}{2}\mathrm{vol}\left(S_{1}^{N-1}\right)\;\chi \left(\Sigma_{v}\right),$$

where $S_{1}^{N-1}$ is an (N−1)-dimensional hypersphere of the unit radius, and $\chi (\Sigma_{v})$ is the Euler characteristic of the level set $\Sigma_{v}$ of the potential function V. $K_{G}$ is the Gauss–Kronecker curvature given by

(76) $$\begin{array}{ccc} K_{G}\left(x\right) & = & \det\left[-\partial_{\beta }\frac{\partial_{\alpha }V}{∥\nabla V∥}+\frac{\partial_{\alpha }V\partial_{\beta }V}{{∥\nabla V∥}^{2}}\right]\\ & = & \det\left[-\frac{\partial_{\alpha }\partial_{\beta }V}{∥\nabla V∥}+\frac{\partial_{\alpha }V\partial_{\beta }\partial_{\mu }V\partial_{\mu }V}{{∥\nabla V∥}^{3}}+\frac{\partial_{\alpha }V\partial_{\beta }V}{{∥\nabla V∥}^{2}}\right]. \end{array}$$

A slight modification of this formula yields the following nontrivial result due to Chern and Lashof [45,46]:

(77) $$\int_{\Sigma_{v}}d\sigma \;\left|K_{G}\right|=\frac{1}{2}\mathrm{vol}\left(S_{1}^{N-1}\right)\sum_{i=0}^{N}\mu_{i}\left(\Sigma_{v}\right)\geq \frac{1}{2}vol\left(S_{1}^{N-1}\right)\sum_{i=0}^{N}b_{i}\left(\Sigma_{v}\right),$$

where $\mu_{i}\left(\Sigma_{v}\right)$ are the Morse indexes of $\Sigma_{v}$, which are defined as the number μ of critical points (∇V=0) of index i on a given level set $\Sigma_{v}$; as already mentioned above, the index i of a critical point is the number of negative eigenvalues of the Hessian of V computed at the critical point. The $b_{i}\left(\Sigma_{v}\right)$ are the Betti numbers of the hypersurfaces. The Betti numbers are fundamental *topological invariants* of differentiable manifolds; they are the diffeomorphism-invariant dimensions of suitable vector spaces (the de Rham cohomology spaces) defined on a given manifold; thus, they are integers.

Another theorem in differential topology that can be constructively used in numerical simulations is Pinkall’s theorem, which states that [47]

(78) $$\int_{\Sigma_{v}}{\left(\sigma^{2}\left(k_{i}\right)\right)}^{n}\;d\eta =Vol\left(S^{n}\right)\sum_{i=1}^{n}{\left(\frac{i}{n-i}\right)}^{n/2-i}b_{i}\left(\Sigma_{v}\right)-r\left(\Sigma_{v}\right),$$

where $d\eta :=d\mu /\int_{\Sigma_{v}}d\mu$, and $Vol(S^{n})$ is the volume of the unit n-sphere, and, given the potential function of a system, $\sigma^{2}\left(k_{i}\right)$ can be easily computed as

(79) $$\begin{array}{cc} \sigma^{2}\left(k_{i}\right) & =\frac{1}{{\left(n-1\right)}^{2}}\left(\frac{Tr[{\left(Hess\;V\right)}^{2}]}{{∥\nabla V∥}^{2}}+\frac{{\langle \nabla V,Hess\;V\;\nabla V\rangle }^{2}}{{∥\nabla V∥}^{6}}-2\frac{{∥Hess\;V\;\nabla V∥}^{2}}{{∥\nabla V∥}^{4}}\right)\\ \; & -\frac{1}{n-1}{\left(\frac{▵V}{∥\nabla V∥}-\frac{\langle \nabla V,Hess\;V\;\nabla V\rangle }{{∥\nabla V∥}^{3}}\right)}^{2} \end{array}$$

where ▵V and HessV are, respectively, the Laplacian and the Hessian of the potential function V, and where $r(\Sigma_{v})$ is a small remainder, we notice that the dispersion of principal curvature is related to the sum of Betti numbers.

Another theorem that can be used is Overholt’s theorem, which states that the range of variability of the scalar curvature can be used to estimate the range of variability of the sectional curvatures, and it is given by [48]

(80) $$\Delta \left(sectional\right)\geq {\left[\frac{vol\left(S_{1}^{N}\right)\sum_{k=0}^{N}b_{k}\left(\Sigma_{v}\right)}{2\;vol(\Sigma_{v})}\right]}^{2/N}.$$

Hence, it turns out that the variations in the topology of $\Sigma_{v}$ detected by the Betti numbers can shape the potential energy profile of Δ(sectional). By being the scalar curvature of a manifold, the sum of all the sectional curvatures, and thereby, the variance of the scalar curvature $R(\Sigma_{v})$, is

(81) $$\sigma_{R}=\frac{\langle R^{2}\left(\Sigma_{v}\right)\rangle -{\langle R\left(\Sigma_{v}\right)\rangle }^{2}}{N(N-1)})≃\Delta \left(sectional\right).$$

#### 4.2.1. Phase Transitions in Small N Systems

A paradigmatic example of a phase transition in small N systems, thus far from the thermodynamic limit, is provided by the protein folding transition, that is, the transition from a filament composed of a sequence of amino acids to the biologically functional folded structure, takes place in systems with a number of degrees of freedom much smaller than the Avogadro number and in the absence of the symmetry-breaking phenomenon. In Ref. [49], a minimalistic model of a polypeptide for the SH3 and PYP protein sequences and for their randomized versions have been considered by means of a C_α_-based Gō-model [50] via the SMOG2 [51] implementation, on the basis of the structures reported in the Protein Data Bank (1FMK for SH3 and 3PHY for PYP). It turns out that in addition to the standard signatures of the folding transition—discriminating between protein sequences of amino acids and random heteropolymer sequences—also peculiar geometric signatures of the equipotential hypersurfaces in configuration space can discriminate between proteins and random heteropolymers. These geometric signatures are the “shadows” of deeper topological changes that take place in correspondence with the protein folding transition. Therefore, seen from the deepest level of topology changes of equipotential submanifolds of the phase space, the protein folding transition fully qualifies as a phase transition, in spite of the small number of degrees of freedom.

**Figure 10:**
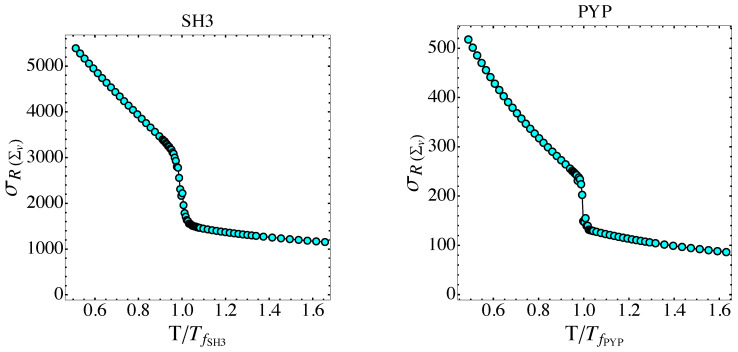
The variance of the scalar curvature of the potential level sets $\Sigma_{v}$ is reported versus temperature T normalized by the folding temperature $T_{f}$ for the SH3 and PYP proteins, respectively.

**Figure 11:**
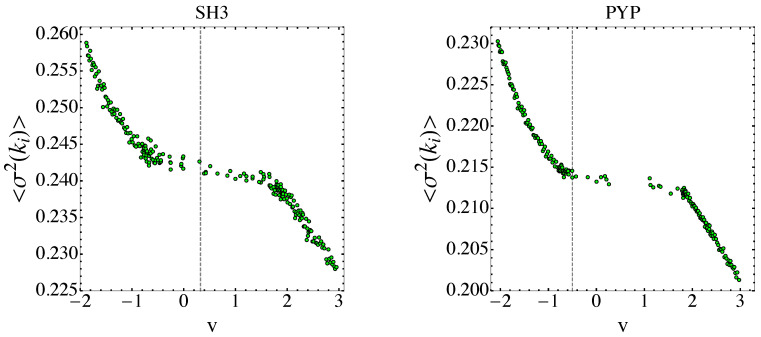
The variance in sectional curvatures of the potential level sets $\Sigma_{v}$ is reported versus potential energy density v for the SH3 and PYP proteins, respectively. Vertical dashed lines correspond to the folding transitions.

In Figure 10, at the folding temperature, the temperature dependence of $\sigma_{R}\left(\Sigma_{v}\right)$ shows a rather sharp jump for the PYP protein and a milder jump with an inflection point for the SH3 protein. After Equations (80) and (81), the underpinning phenomenon of the temperature dependence of $\sigma_{R}\left(\Sigma_{v}\right)$ is a jump in the sum of the Betti numbers, and thus a major change in the topology of the potential level sets $\Sigma_{v}$. This is confirmed by Figure 11, where the potential energy density dependence of $\langle \sigma^{2}\left(k_{i}\right)\rangle$ is reported. In fact, according to the Pinkall theorem expressed by Equation (78), the jumps of $\langle \sigma^{2}\left(k_{i}\right)\rangle$ versus v are closely related to the corresponding jumps of a weighted sum of Betti numbers.

#### 4.2.2. Phase Transitions in the Absence of Symmetry Breaking

On a 3d-lattice Λ, with I={(1,0,0),(0,1,0),(0,0,1)}, the following model Hamiltonian describes a dual Ising model [52] added with a quadratic kinetic energy term and a quartic term introduced for numerical reasons:

(82) $$H\left(\pi ,U\right)=\sum_{i\in \Lambda }\sum_{\mu \in I}\frac{1}{2}\pi_{i\mu }^{2}-J\sum_{□\in \Lambda }U_{ij}U_{jk}U_{kl}U_{li}+\alpha \sum_{i\in \Lambda }\sum_{\mu \in I}{\left(U_{i\mu }^{2}-1\right)}^{4}\;.$$

**Figure 12:**
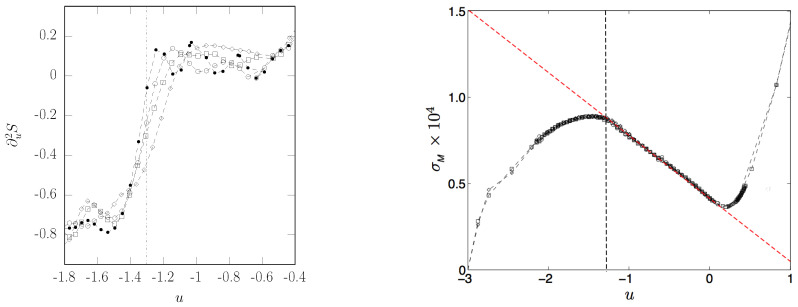
**Left panel**: Second derivative $\partial^{2}S/\partial u^{2}$ of the configurational entropy versus the average potential energy per degree of freedom u. Lattice dimensions: $n^{3}=6\times 6\times 6$ (rhombs), $n^{3}=8\times 8\times 8$ (squares), $n^{3}=10\times 10\times 10$ (circles), $n^{3}=14\times 14\times 14$ (full circles). The vertical dot-dashed line locates the phase transition point. **Right panel**: second moment of the total mean curvature of the potential level sets $\Sigma_{u}$ versus the average potential energy per degree of freedom u. $n^{3}=6\times 6\times 6$ (rhombs), $n^{3}=8\times 8\times 8$ (squares), $n^{3}=10\times 10\times 10$ (circles). The oblique dashed line is a guide to the eye. The vertical dashed line at u≃−1.32 corresponds to the phase transition point and to the point where the second derivative $d^{2}\sigma_{M}/du^{2}$ jumps from a negative value to zero.

This model undergoes a phase transition but, having a gauge (local) symmetry

$$U_{ij}\to \varepsilon_{i}\varepsilon_{j}U_{ij}\;\varepsilon_{i},\varepsilon_{j}=\pm 1,\;i,j\in \Lambda$$

after Elitzur’s theorem [53], it has no order parameter, and the phase transition is not associated with a global symmetry breaking mechanism. The numerical integration of the Hamilton equations of motion of this model allows to detect a first-order phase transition through thermodynamic observables [41]. Among the other observables detecting the transition, in Figure 12, the second-order derivative (with respect to the potential energy density) of the configurational entropy displays a jump which steepens at increasing N (left panel). Correspondingly, the right panel of Figure 12 displays a clear jump in the second-order derivative (with respect to the potential energy density) of the quantity denoted by $\sigma_{M}\left(v\right)$, the variance of the mean curvature integrated over a potential level set $\Sigma_{v}$, which is explicitly related to the topology of these level sets after the above-mentioned Pinkall’s theorem [41].

#### 4.2.3. Prospective Applications to Phase Transitions in Quantum Systems

In order to extend to quantum systems the geometric and topological methods developed for classical systems, among other possibilities sketched in [16], we highlight the possible use of the Time-Dependent Variational Principle (TDVP) technique [54,55]. The same technique, more deepened from both the mathematical and conceptual points of view, has been proposed as a “dequantization” technique in [56] as a kind of inverse procedure with respect to the geometrical quantization. This consists in making the ansatz that the wavefunction of a system depends on N parameters ${\left\{x_{i}\right\}}_{i=1,...,N}$

(83) $$|\psi \rangle =|\psi (x_{1},...,x_{N})\rangle .$$

where the parameters $x_{i}=x_{i}\left(t\right)$ are, in general, functions of time. For a quantum system with Hamiltonian ${\hat{H}}_{Tot}$, the equations of motion of the $x_{i}$ can be derived using the following variational principle (equivalent to the least action principle):

(84) $$\delta S=0\;\mathrm{with}\;S=\int_{0}^{t}L\left(\psi ,\bar{\psi }\right)\;\;dt^{\prime }$$

where L(ψ,) is the Lagrangian associated to the system

(85) $$L\left(\psi ,\bar{\psi }\right)=\frac{ı\hbar }{2}\frac{\langle \psi |\dot{\psi }\rangle -\langle \dot{\psi }|\psi \rangle }{\langle \psi |\psi \rangle }-\frac{\langle \psi |{\hat{H}}_{Tot}|\psi \rangle }{\langle \psi |\psi \rangle }.$$

The equations of motions derived from Equation (84) can be worked out in the framework of classical Hamiltonian dynamics.

The classical Hamiltonian is associated with the quantum one by simply taking the expectation value of the Hamiltonian operator ${\hat{H}}_{tot}$ over the state $|\psi (x_{1},...,x_{N})\rangle$, that is

(86) $$H_{Tot}=\langle \psi (x_{1},...,x_{N})|{\hat{H}}_{Tot}|\psi (x_{1},...,x_{N})\rangle .$$

It is worth mentioning that the TDVP, being a variational approach, applies generically to any quantum system and its effectiveness depends on a reasonable choice of the initial ansatz for the state vector. Moreover, the remarkable fact is that the dynamical equations worked out by means of the TDVP are formally classical but give the time evolution of actual quantum expectation values. Therefore, this seems a promising way to study also quantum phase transitions in the above outlined topological framework.

Another potential method to extend the geometrical approach to quantum systems, particularly quantum field theories on a lattice, is based on the Wick correspondence between the path-integral approach to quantum field theory and classical statistical mechanics. Recent findings indicate that a microcanonical description of quantum field theory [57] on a lattice provides new insights into the deconfinement transition in lattice quantum field theory [58]. This conceptual framework is highly suitable for applying the geometrical and topological characterizations of phase transitions.

## 5. Conclusions

Phase transition phenomena are associated with apparently singular (nonanalytical) behaviors of the experimentally detected thermodynamic observables at the transition point. This is at odds with the analytical property of the statistical measures that are used to theoretically compute (at least in principle) all the thermodynamic observables. The problem was fixed by the Yang–Lee theorem, which led to the so-called *thermodynamic limit—(N→∞) dogma*. Since then, the way we explain the origin of singular behaviors of thermodynamic observables has been identified with the physical phenomenon. In other words, one can properly speak of phase transitions only for systems with an infinite number of degrees of freedom. However, not only does the Avogadro number have nothing to do with N=∞,but modern research in the field has shown a broad variety of experimentally detected phase transitions taking place in small N systems. A snowflake of a few hundreds of water molecules suddenly melting to a nanoscopic droplet of water [59], a Bose–Einstein condensate of a few hundreds of atoms [60], a superconductive nanoparticle levitating in a magnetic field that suddenly falls down when the temperature is raised, a filament-globule transition in homopolymers [61], and a protein folding transition [62,63,64] are sudden qualitative changes in the physical properties of small N systems, at odds with the theoretical thermodynamic limit dogma.

Moreover, in the Landau theory, phase transitions are usually associated with the phenomenon of a spontaneous symmetry breaking, that is, at low temperatures, the accessible states of a system can be missing some of the global symmetries of the Hamiltonian, and thus, the corresponding phase is the less symmetric one; to the contrary, at higher temperatures, a wider range of energy states having all the symmetries of the Hamiltonian are made accessible by the thermal fluctuations. In the symmetry-breaking phenomena, the physical states of a system are characterized by the order parameter, an extra thermodynamic observable. The order parameter vanishes in the symmetric phase and is different from zero in the broken-symmetry phase. But there are many phase transitions that lack an order parameter and are not associated with the symmetry-breaking phenomenon as is the case of two-dimensional systems with continuous symmetry, after the Mermin–Wagner theorem [65,66], and systems with gauge symmetry, after the Elitzur theorem [53].

The study of phase transitions from a topological point of view has been carried out for several kinds of systems; the nonexhaustive list ranges from entropy-driven transitions [67,68] (having also applications to robotics), to quantum phase transitions [69,70,71], glasses and supercooled liquids [72,73], classical models in statistical mechanics [74,75,76], discrete spin models [77], DNA denaturation [78], and peptide structure [79]. Actually, let us remark that before the explicit formulation of the topological hypothesis [7,8], in many contexts, topological concepts have already and implicitly entered the study of phase transitions while referring to energy landscapes [80,81] and to saddle points of the potential energy of disordered systems, glasses, and spin glasses [72,73]; in fact saddle points are critical points in the language of the Morse theory of differential topology.In reference [82], a topological phase transitions in functional brain networks is reported by resorting to concepts from topological data analysis, topology, geometry, physics, and network theory. The turning point qualifying all the above-mentioned results as a topological theory of phase transitions can be identified with the necessity theorem outlined in Section 4.1.2.

The topological theory of phase transitions provides a unifying framework to tackle phase transitions that may or may not be due to a symmetry-breaking phenomenon (that is, with or without an order parameter) and to finite/small N systems. The conceptual leap consists in having unveiled a deeper origin of the phenomenon with respect to its standard description through the singularities of statistical measures. In fact, phase transitions are necessarily rooted in the loss of diffeomorphicity at $v_{c}$—the critical value of the average potential energy density at which the phase transition takes place—among the members of the family ${\left\{\Sigma_{v}\right\}}_{v<v_{c}}$ and those of the family ${\left\{\Sigma_{v}\right\}}_{v>v_{c}}$, as well as among the members of the family ${\left\{M_{v}\right\}}_{v<v_{c}}$ and those of ${\left\{M_{v}\right\}}_{v>v_{c}}$. But these manifolds depend only on the knowledge of the potential energy of a system, that is, on the interaction potential among its degrees of freedom: nothing else.

There is still room for extensions of the theory, mainly from the computational viewpoint regarding sufficiency conditions, as well as its extension to quantum phase transitions.

## Figures and Tables

**Figure 3:**
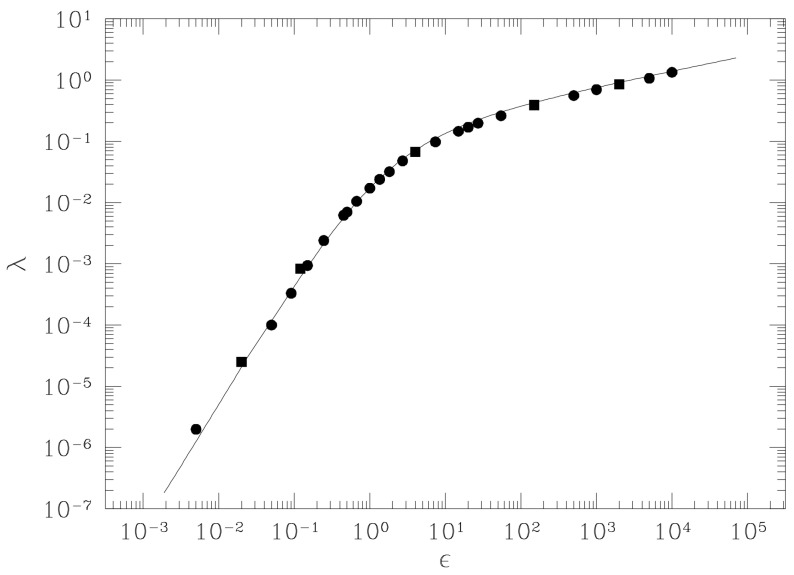
Lyapunov exponent λ vs. energy density ε for the FPU β model with β=0.1. The continuous line is the theoretical computation according to Equation (56), while the circles and squares are the results of numerical simulations with N respectively equal to 256 and 2000. From [31].
